# Analgesic effectiveness of serratus anterior plane block in patients undergoing video-assisted thoracoscopic surgery: a systematic review and updated meta-analysis of randomized controlled trials

**DOI:** 10.1186/s12871-023-02197-8

**Published:** 2023-07-13

**Authors:** Jie Li, Xiaoyu Wang, Yinge Wang, Wenwu Zhang

**Affiliations:** Department of Anesthesiology, Yuncheng Central Hospital, Shanxi Medical University, Yuncheng, 044000 Shanxi Province China

**Keywords:** Serratus anterior plane block, Video-assisted thoracoscopic surgery, Analgesia, Postoperative pain scores, Meta-analysis

## Abstract

**Background:**

Serratus anterior plane block (SAPB) is a promising regional technique for analgesia in thoracic surgery. Till now, several randomized controlled trials (RCTs) have explored the effectiveness of SAPB for postoperative pain control in patients undergoing video-assisted thoracoscopic surgery (VATS), but the sample sizes were small and conclusions remained in controversy. Therefore, we conducted the present systematic review and meta-analysis.

**Methods:**

RCTs evaluating the analgesic performance of SAPB, comparing to control methods (no block, placebo or local infiltration anesthesia), in patients undergoing VATS were searched in PubMed, EMBASE, Web of Science and Cochrane Library from inception to December 31, 2022. Mean difference (MD) and corresponding 95% confidence interval (95%CI) were calculated for postoperative pain scores at various time points, postoperative opioid consumption and length of hospital stay. Pooled relative risk (RR) with 95%CI were calculated for the risk of postoperative nausea and vomiting (PONV) and dizziness. A random-effect model was applied.

**Results:**

A total of 12 RCTs (837 participants) were finally included. Compared to control group, SAPB had significant reductions of postoperative pain scores at 2 h (MD = -1.58, 95%CI: -1.86 to -1.31, *P* < 0.001), 6 h (MD = -2.06, 95%CI: -2.74 to -1.38, *P* < 0.001), 12 h (MD = -1.72, 95%CI: -2.30 to -1.14, *P* < 0.001) and 24 h (MD = -1.03, 95%CI: -1.55 to -0.52, *P* < 0.001), respectively. Moreover, SAPB conferred a fewer postoperative opioid consumption (MD = -7.3 mg of intravenous morphine equivalent, 95%CI: -10.16 to -4.44, *P* < 0.001) and lower incidence of PONV (RR = 0.56, 95%CI: 0.41 to 0.77, *P* < 0.001). There was no difference between both groups regarding length of hospital stay and risk of dizziness.

**Conclusion:**

SAPB shows an excellent performance in postoperative pain management in patients undergoing VATS by reducing pains scores, postoperative opioid consumption and incidence of PONV. However, due to huge heterogeneity, more well-designed, large-scale RCTs are needed to verify these findings in the future.

**Supplementary Information:**

The online version contains supplementary material available at 10.1186/s12871-023-02197-8.

## Introduction

Patients undergoing thoracic surgery frequently experience acute and chronic postoperative pain [1]. Insufficient postoperative pain control will delay the recovery of pulmonary function, increase the length of hospital stay, and lead to postoperative complications such as pulmonary infection, which is negatively related to quality of life and patient’s satisfaction [2, 3]. Thus, postoperative acute pain management is a very important issue for patients suffering postoperative pain. Video-assisted thoracoscopic surgery (VATS), using a smaller incision, is less invasive and confers less postoperative pain than the traditional open thoracotomy [4, 5]. Thus, VAST has now been widely accepted and become the gold standard for many thoracic surgeries [6]. Despite of these advantages, a proportion of patients receiving VATS still experience moderate to severe pain after surgery, and achievement of postoperative analgesia is still challenging [7].

Opioids are commonly used drugs, given intravenously, orally or via patient-controlled analgesia (PCA) devices, for postoperative pain management [8]. However, opioid-related side effects are quite considerable, including nausea, vomiting, hypotension, sedation and respiratory depression [9, 10]. Consequently, a multimodal perioperative analgesia, combining intravenous analgesia and regional nerve block, has been proposed to lessen opioid consumption and achieve a better pain control [11]. Several widely adopted regional blocks, in the past decades, include thoracic epidural analgesia (TEA), intercostal nerve blockade (ICNB) and thoracic paravertebral blockade (TPVB) [12, 13]. Yet, these regional blocks are technically more challenging and may have potential complications and side effects [14].

Serratus anterior plane block (SAPB), a ultrasound-guided region block technique was firstly proposed by Blanco et al. [15]. This technique, injecting a certain concentration and volume of non-opioid analgesics into the surface of the anterior serratus muscle or deep interstitial space by a simple operation, confers a more complete and wider nerve block effect and has less complications [15]. SAPB has shown good performance on postoperative pain management and reducing postoperative opioid consumption in thoracotomy, breast surgery and rib fracture surgery [16–18]. Thus, SAPB appears to be an effective and safe region block that is easy to perform.

However, current evidence of the postoperative analgesic effect of SAPB on patients undergoing VATS is still limited. Several randomized controlled trials (RCTs) have been performed, but had small sample sizes and yielded inconsistent results [19]. Therefore, we performed a systematic review and meta-analysis of RCTs, to evaluate the analgesic effectiveness of SAPB after VATS in terms of postoperative pain scores, postoperative opioid consumption and adverse reactions.

## Methods

### Literature search

This is a systematic review and meta-analysis of randomized controlled trials (RCTs) performed in accordance with the Preferred Reporting Items for Systematic Review and Meta-analysis (PRISMA). The PRISMA checklist was shown in Additional file 1. We searched PubMed, EMBASE, Web of Science and Cochrane Library for candidate studies from inception to December 31, 2022. The search strategy for each electronic database was listed in Additional file 2. There was no language restriction. Additional articles were obtained by manually checking to reference lists of eligible studies and reviews related to the topic.

### Inclusion and exclusion criteria

Two independent researchers filtered articles retrieved from literature search by viewing titles and abstracts, and further obtained eligible studies by reading the full texts. All studies were included according to the PICOS framework: Population (P): adult patients undergoing any type of VATS; Intervention (I): single-shot SAPB; Control (C): no block or placebo, with or without would infiltration; Outcome (O): postoperative pain scores at rest, postoperative opioid consumption, postoperative nausea and vomiting (PONV), dizziness, and length of hospital stay; Study design (S): RCT. Those studies comparing SAPB to other regional analgesia blocks or using continuous SAPB were excluded. Case reports, reviews and studies without sufficient data were discarded.

### Outcomes

The primary outcome was the postoperative pain scores at different time points (2, 6, 12 and 24 h) and the postoperative opioid consumption during the first 24 h after surgery. The secondary outcomes included the incidences of PONV and dizziness and the length of hospital stay.

### Data extraction

The following information of each included trial was extracted by two independent researchers: first author, publication year, sample size, SAPB type (superficial or deep), sample size, age, weight, percentage of males, American Society of Anesthesiology (ASA) class, SAPB regimen, concomitant pain management. All different opioid consumption was converted to intravenous morphine equivalents using conversion tool from GlobalRPh website (https://www.globalrph.com/narcotic) assuming 0% incomplete cross tolerance.

### Risk of bias, quality assessment and certainty of evidence

The risk of bias of each study was assessed according to Cochrane Collaboration’s tool for assessing risk of bias. This tool includes several domains, i.e. selection bias (random sequence generation, allocation concealment), performance bias (blinding of participants and personnel), detection bias (blinding of outcome assessment), attrition bias (incomplete outcome data), reporting bias (selective reporting) and other bias. Each domain was graded as low, unclear or high risk of bias. Moreover, the quality of included studies was assessed by using JADAD scale [20]. The JADAD scale assigned 0, 1 or 2 scores to three domains regarding randomization, blinding and withdrawals and dropouts according to the description and appropriateness of these domains. A study with a total score of 3–5 was considered to be of high quality; otherwise it was of low quality. The level of certainty of evidence was assessed by using Grading of Recommendations Assessment, Development and Evaluation (GRADE) approach [21]. The literature search, data extraction, risk of bias and quality assessment were conducted by two independent researchers, and agreements were reached by further discussion if there were conflicts.

### Statistical analysis

We performed this meta-analysis by using STATA 16.0 (Stata Corporation, TX, USA). Between-study heterogeneity was assessed by *I*^*2*^ statistic. *I*^*2*^ < 50% with P value of Q test > 0.1 indicated low heterogeneity, and a fixed-effect model was applied. Otherwise, a random-effect model was used. For continuous variables (postoperative pain scores, postoperative opioid consumption, length of hospital stay), mean difference (MD) and corresponding 95% confidence interval (95%CI) were calculated. For categorical variables (PONV, dizziness), relative risk (RR) and 95%CI were calculated. In addition, subgroup analyses of superficial SAPB and deep SAPB, and a “leave-one-out” sensitivity analysis were performed. Further sensitivity analysis excluding studies having high or unclear risk of bias in at least one domain according to Cochrane assessment or having a low quality according to JADAD scale was performed. Publication bias was evaluated by Egger’s test. *P* value less than 0.05 indicated statistical significance.

## Results

### Baseline characteristics of eligible studies included in meta-analysis

**Fig. 1:**
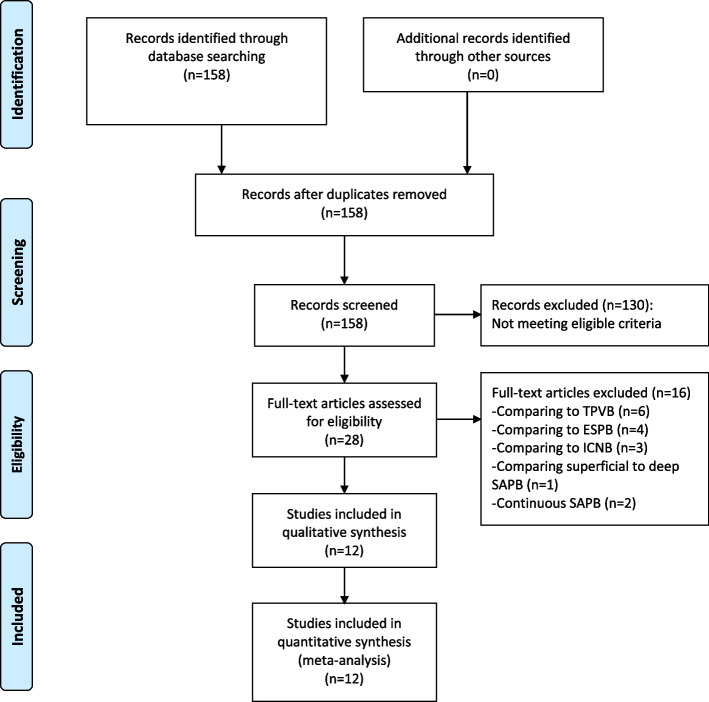
Flowchart of literature search

We obtained 158 articles from electronic databases by literature search, of which 130 articles that were reviews, case reports, unrelated to the topic or not RCT-designed were discarded by viewing titles and abstracts. Of the remaining 28 articles, 16 were excluded due to the following reasons: 6 comparing SAPB to TPVB, 4 comparing SAPB to erector spinae plane block (ESPB), 3 comparing SAPB to ICNB, 1 comparing superficial to deep SAPB, 2 investigating continuous SAPB. As illustrated in Fig. 1, a total of 12 eligible RCTs involving 837 participants were finally included for the meta-analysis [19, 22–32].

Ropivacaine was used for regional block in 9 RCTs, and the concentrations ranged from 0.25% to 0.50% [22–28, 30, 31]. Bupivacaine was used in 3 RCTs with a concentration of 0.25% [19, 29, 32]. As to the control group, 8 trials had no block [22–24, 26, 28–30, 32], 1 used normal saline as placebo [31], 3 adopted local infiltration anesthesia [19, 25, 27]. In addition to regional nerve block, postoperative pain control included patient-controlled analgesia (PCA) given to all participants in 9 trials [19, 22–25, 27, 30–32] and standard pain management in the other 3 trials [26, 28, 29]. A half of the trials enrolled patients meeting the criteria of classes I-II of the American Society of Anesthesiology (ASA) [19, 22, 27, 28, 30, 31], and the rest recruited ASA class I-III patients. SAPB can be divided into two types, i.e. superficial SAPB targeting the interfascial plane between the latissimus dorsi and serratus anterior muscle, and deep SAPB injecting block regimen between serratus anterior and intercostal muscle. Among the included trials, 3 adopted only deep SAPB [26, 30, 32] and 1 used both techniques [29]. Another study assigned patients to superficial SAPB group and deep SAPB group [24]. There, control patients in this study should be split into 2 groups to avoid adding extra 21control patients that do not exist as previously reported [33]. Briefly, the number of participants were divided with mean and SD unchanged for continuous outcomes, and the number of events and total number were both divided for dichotomous outcomes. The rest applied only superficial SAPB. The characteristics of all trial included in the meta-analysis were summarized in Table 1.

**Table 1:** Characteristics of studies included in the meta-analysis

Author	Year	Sample	Age, years	Weight, kg	Male%	SAPB	Control	Concomitant pain control	JADAD scale
Okmen	2018	20/20	53.5 ± 8.67/54.5 ± 7.92	75.25 ± 10.99/73.7 ± 10.4	55/50	20 ml of 0.25% bupivacaine	No block	PCA (tramadol)	4
Kim	2018	42/43	56.4 ± 8.7/54.7 ± 8.7	60.6 ± 10.3/63.4 ± 10.8	33.3/32.6	0.4 ml/kg of 0.375% ropivacaine	Normal saline	PCA (fentanyl)	5
Park	2018	42/42	58.0 ± 9.4/58.4 ± 10.5	63.8 ± 9.9/63.9 ± 12.0	40/43	30 ml of 0.375% ropivacaine	No block	PCA (fentanyl)	5
Semynonov	2019	47/57	62 ± 14.857/56.1 ± 17.83	72.3/71.8	44/29.8	2 ml/kg of 0.25% bupivacaine hydrochloride	No block	Standard pain management	2
Lee	2019	23/23	63.6 ± 9.9/65.1 ± 8.4	63.3 ± 12.3/61.7 ± 10.2	61/65	20 ml of 0.375% ropivacaine	No block	Standard pain management	5
Chen	2019	20/20	58.9 ± 5.7/57.1 ± 6.2	63.9 ± 7.8/62.3 ± 8.8	55/60	0.4 ml/kg of 0.25% ropivacaine	Local anesthetic infiltration	PCA (sufentanil)	4
Viti	2020	46/44	67.8 ± 9.3/71 ± 7.9	-	60.7/68.2	30 ml of 0.3% ropivacaine	No block	Standard pain management	5
Shang	2020	30/30	56.2 ± 7.2/58.23 ± 9.03	-	50/57	20 ml of 0.5% ropivacaine	Local anesthetic infiltration	PCA (butorphanol)	5
Qiu^a^	2021	21/21/21	62.7 ± 8.1/63.0 ± 8.9/64.9 ± 8.3	62.4 ± 11.7/64.6 ± 7.4/60.8 ± 8.7	52.4/61.9/42.9	0.4 ml/kg of 0.375% ropivacaine	No block	PCA (sufentanil)	4
Er	2021	39/38	53.47 ± 11.63/52.36 ± 10.87	-	53.8/60.5	15 ml of 0.375% ropivacaine	No block	PCA (sufentanil)	5
Dikici	2022	30/30	53.2 ± 14.5/52.4 ± 14.3	-	46.7/46.7	0.25 ml/kg of 0.25% bupivacaine	Local anesthetic infiltration	PCA (morphine hydrochloride)	3
Liu	2022	44/44	59.16 ± 9.68/58.95 ± 9.64	-	63.6/59.1	20 ml of 0.375% ropivacaine	No block	PCA (sufentanil)	3

*SAPB* serratus anterior plane block, *PCA* patient-controlled analgesia

^a^Including 3 groups: superficial SAPB, deep SAPB and control group

### Risk of bias and study quality

**Fig. 2:**
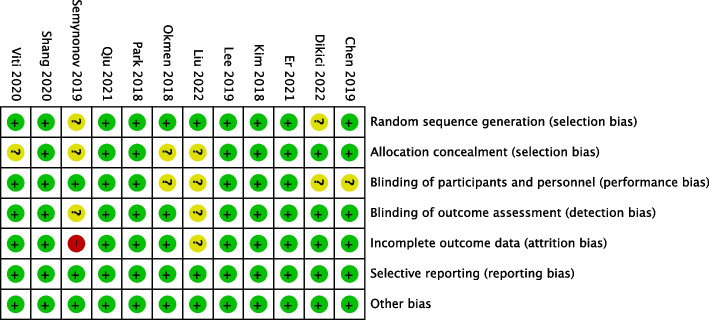
Risk of bias summary

**Fig. 3:**
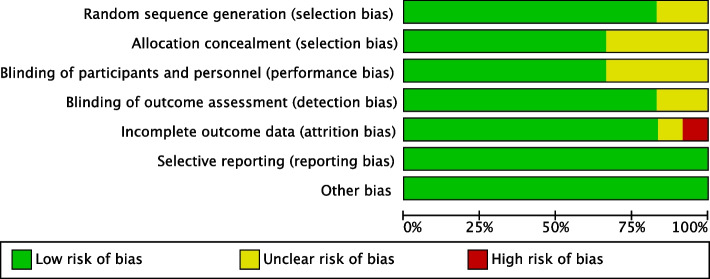
Risk of bias graph

Ten trials reported appropriated randomization procedures, most of which adopted a computer-generated randomization sequence [22–28, 30–32]. Eight trials described allocation concealment [19, 23–25, 27, 28, 30, 31]. The blind method for participants and personnel was not reported in 4 trials [19, 22, 27, 32]. In 10 trials, the investigators assessing the postoperative parameters were blinded to the group assignment and the surgery procedure [19, 23–28, 30–32]. Taken together, 6 studies had low risk of bias in all domains [23–25, 28, 30, 31]. The risk of bias assessment was summarized in Fig. 2 and Fig. 3. According to JADAD scale, one study with 2 scores was considered to have a low quality [29], and the others with 3 to 5 scores had a high quality.

### Postoperative pain scores

**Fig. 4:**
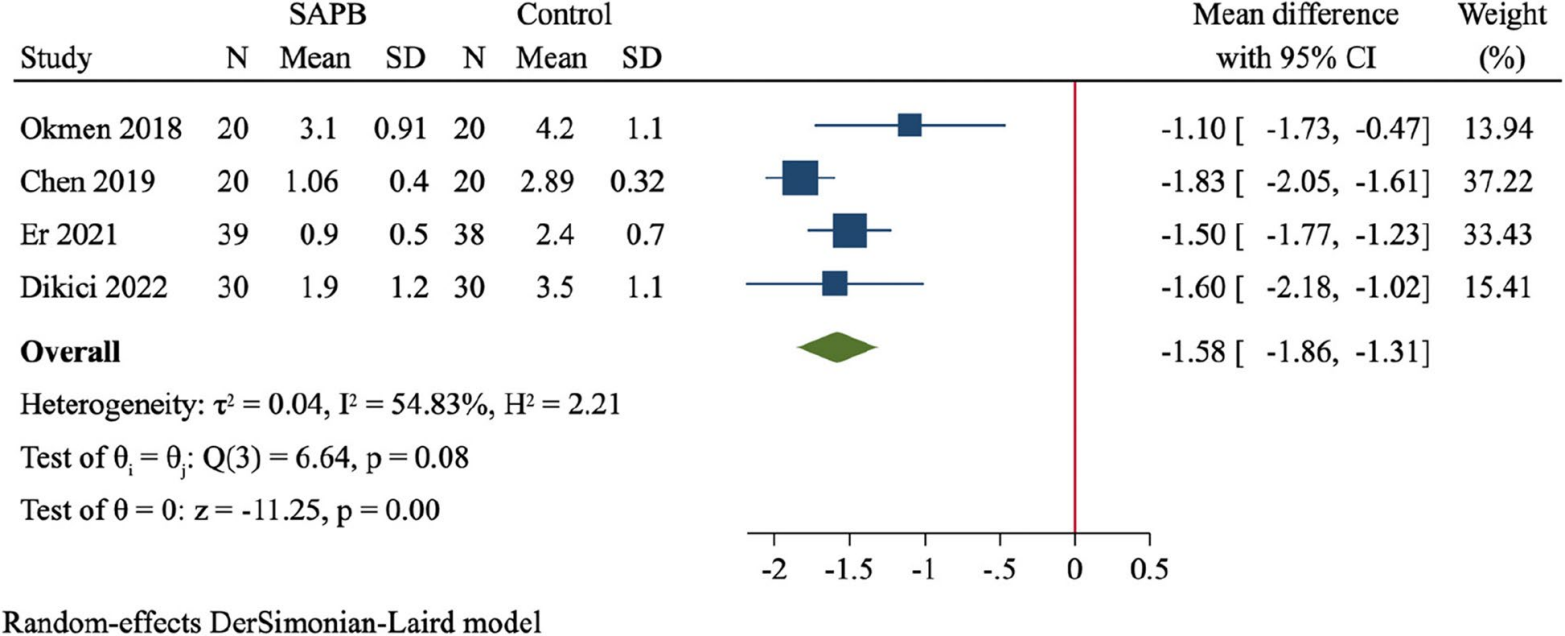
Forest plot for meta-analysis of postoperative pain scores at 2 h

Four studies, including 109 patients in SAPB group and 109 in control group, assessed the pain score at 2 h after surgery [19, 23, 27, 32]. There was substantial between-study heterogeneity (*I*^*2*^ = 54.8%). SAPB had a significant improvement of postoperative pain score compared to control (MD = -1.58, 95%CI: -1.86 to -1.31, *P* < 0.001, Fig. 4).

**Fig. 5:**
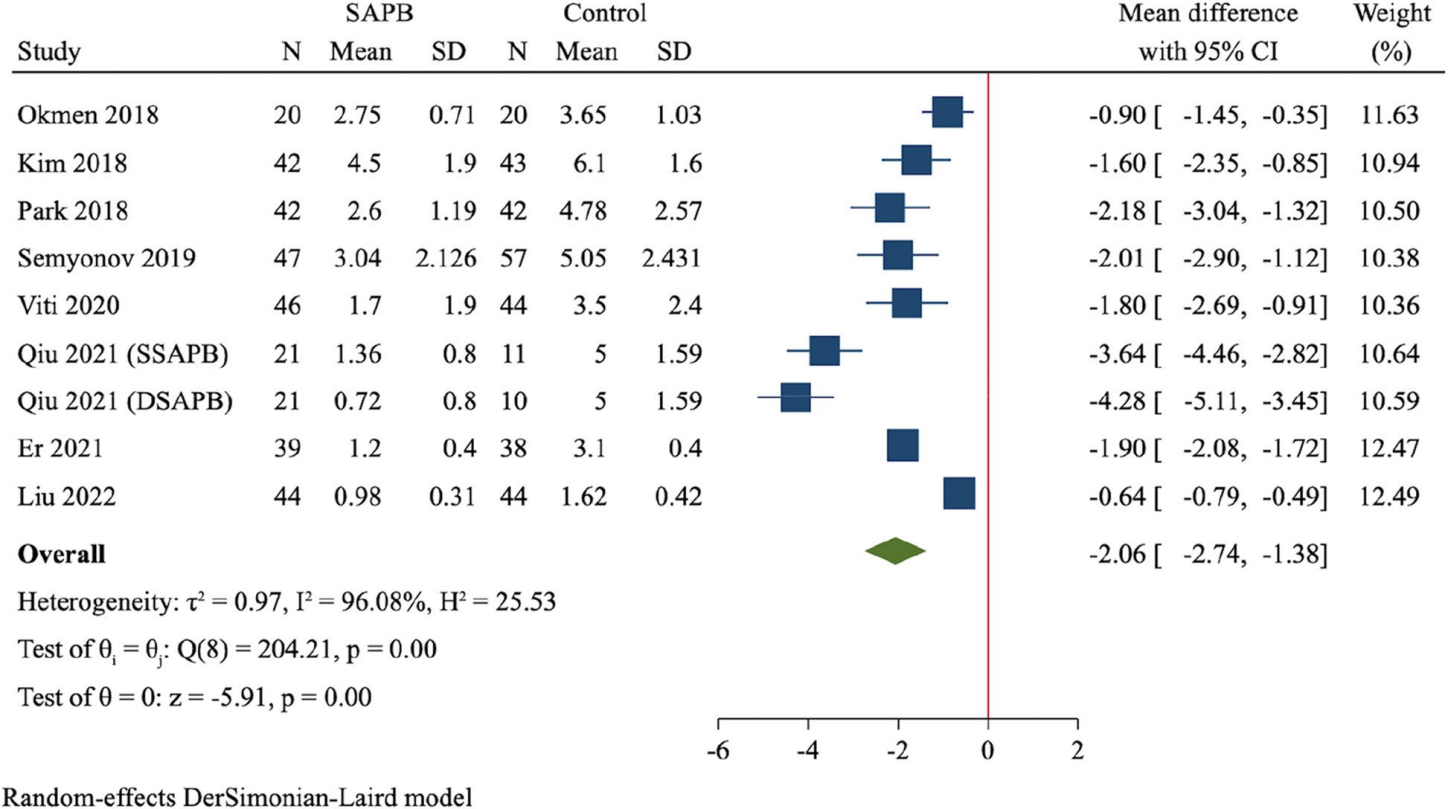
Forest plot for meta-analysis of postoperative pain scores at 6 h

The postoperative pain score at 6 h were reported in 9 trials, which assigned 322 patients to SAPB group and 309 to control group [22–24, 26, 29–32]. Meta-analysis using a random-effect model showed a significant reduction of pain score at 6 h after surgery in SAPB group compared to control group (MD = -2.06, 95%CI: -2.74 to -1.38, *P* < 0.001, Fig. 5).

**Fig. 6:**
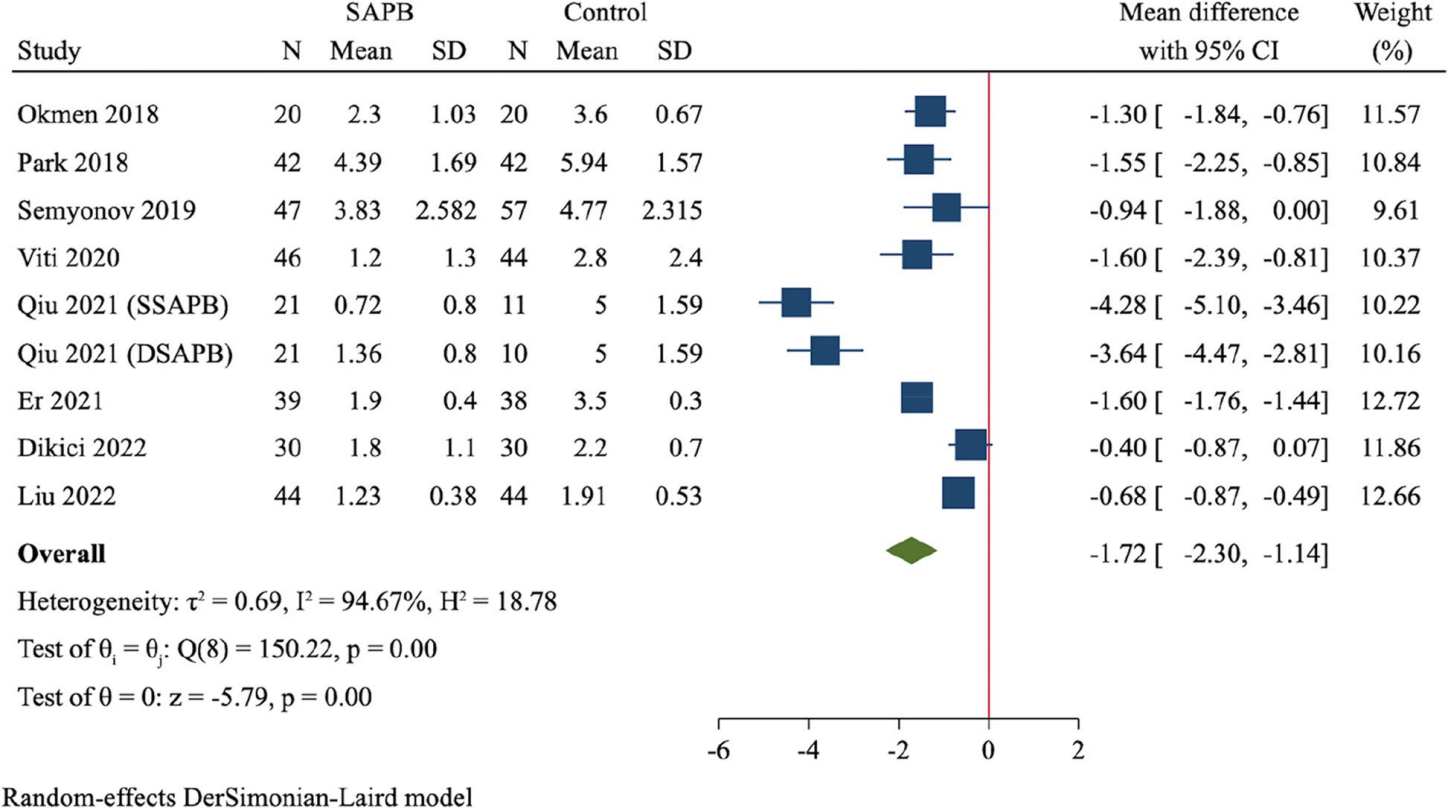
Forest plot for meta-analysis of postoperative pain scores at 12 h

We pooled 9 trials, including 310 and 296 participants in SAPB and control group, respectively, for the evaluation of pain scores at 12 h after surgery [19, 22–24, 26, 29, 30, 32]. Compared to control group, SAPB had a significantly lower postoperative pain score at 12 h (MD = -1.72, 95%CI: -2.30 to -1.14, *P* < 0.001, Fig. 6).

**Fig. 7:**
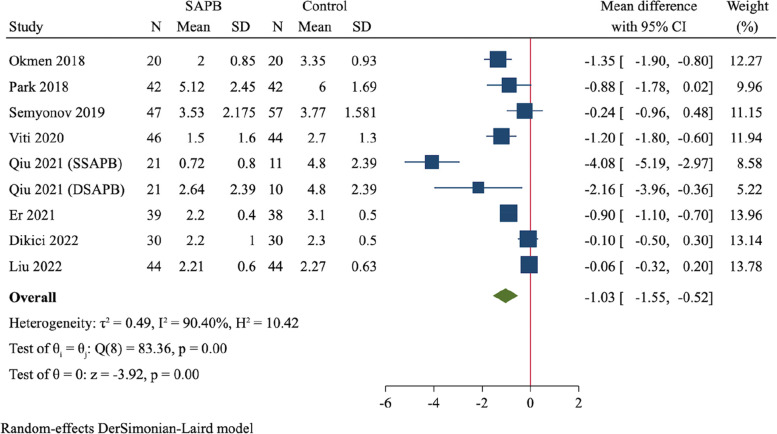
Forest plot for meta-analysis of postoperative pain scores at 24 h

Nine RCTs, including 310 patients in SAPB group and 296 patients in control group, evaluated the postoperative pain scores at 24 h [19, 22–24, 26, 29, 30, 32]. Pooled analysis demonstrated a significantly lower pain scores in SAPB group than control group (MD = -1.03, 95%CI: -1.55 to -0.52, *P* < 0.001, Fig. 7).

Subgroup analysis of pain scores according to different SAPB techniques at 6, 12 and 24 h after surgery were performed (Table 2). At each time point, both superficial and deep SAPB showed significantly lower pain scores than control group. However, between-subgroup comparison showed no significant difference of pain score reduction between both subgroups (*P* > 0.05).

**Table 2:** Subgroup analyses of postoperative pain scores according to SAPB types

SAPB type	Postoperative time point, hours	No. of studies	Sample	*I*^*2*^ (%)	MD	95%CI	*P*
Superficial SAPB	6	4	146/146	98.0	-1.90	-2.88 to -0.92	< 0.001
	12	4	134/133	97.6	-1.66	-2.55 to -0.77	< 0.001
	24	4	134/133	95.8	-1.06	-1.89 to -0.24	0.012
Deep SAPB	6	4	129/127	94.0	-2.28	-3.81 to -0.75	0.003
	12	4	129/127	88.6	-2.01	-3.03 to -0.99	< 0.001
	24	4	129/127	0	-1.27	-1.63 to -0.91	< 0.001

*SAPB* serratus anterior plane block, *MD* mean difference; CI: confidence interval

### Postoperative opioid consumption

**Fig. 8:**
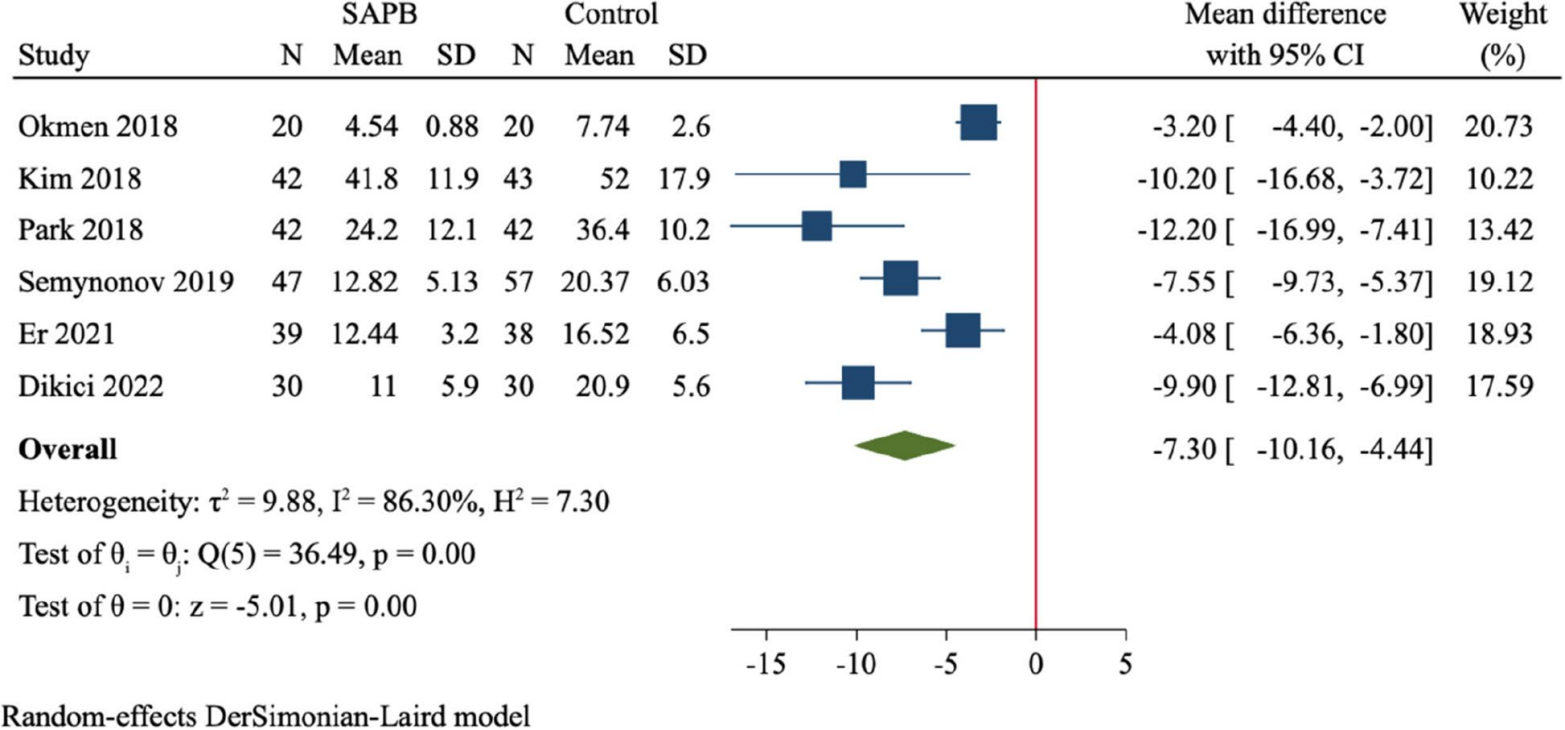
Forest plot for meta-analysis of postoperative opioid consumption

The cumulative opioid consumption within the first 24 h after surgery was assessed in 6 trials including 220 and 230 participants in SAPB and control group, respectively [19, 23, 29–32]. There was substantial between-study heterogeneity (*I*^*2*^ = 86.3%), and a random-effect model was applied. Compared to control, SAPB had a significantly lower postoperative opioid consumption (MD = -7.30 mg of intravenous morphine equivalent, 95%CI: -10.16 to -4.44, *P* < 0.001, Fig. 8).

### Length of hospital stay

The length of hospital stay was recorded in 6 trials, which included 233 cases in SAPB group and 231 cases in control group [22, 23, 26, 27, 30, 31]. Pooled analysis showed no significant difference of length of hospital stay between both groups (MD = -1.07 days, 95%CI: -2.41 to 0.27, *P* = 0.118, Additional file 3).

#### PONV

**Fig. 9:**
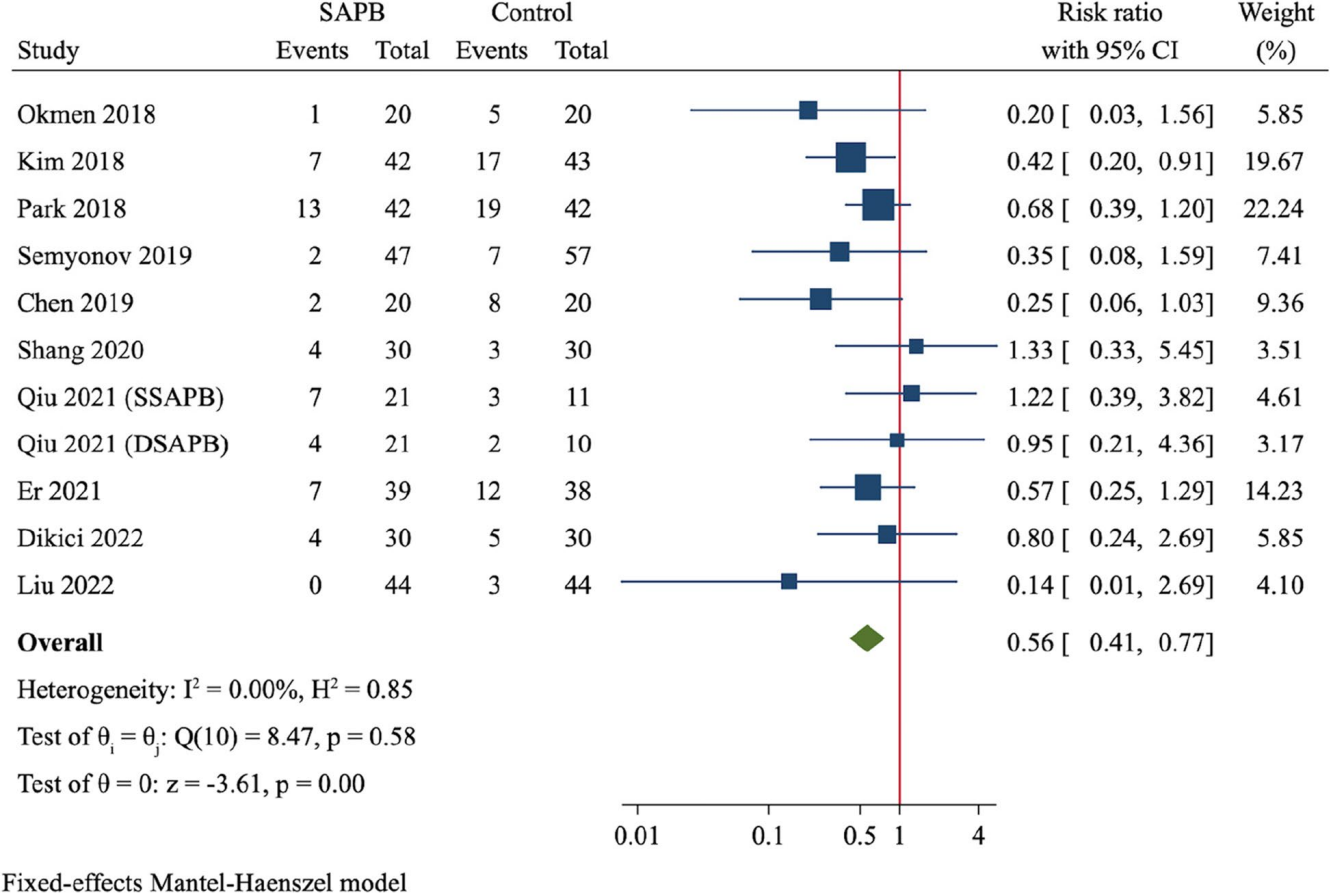
Forest plot for meta-analysis of postoperative nausea and vomiting

The incidence of PONV was reported in 11 trials [19, 22–25, 27, 29–32]. The overall incidences of PONV were 14.3% (51/356) in SAPB group and 24.3% (84/345) in control group, respectively. There was no between-study heterogeneity (*I*^*2*^ = 0), and the fixed-effect model was applied. Pooled analysis demonstrated a significantly reduced risk of PONV (RR = 0.56, 95%CI: 0.41 to 0.77, *P* < 0.001, Fig. 9) in SAPB group than control group.

### Dizziness

The incidences of dizziness were 7.2% (12/167) in SAPB group and 12.0% (20/167) in control group, respectively, which showed no significant difference (RR = 0.60, 95%CI: 0.31–1.16, *P* = 0.130, Additional file 4).

### Sensitivity analysis, publication bias and certainty of evidence

Sensitivity analysis using the “leave-one-out” method suggested that the omission of any study did not had significant impact on the pooled results. Further sensitivity analysis was conducted by only pooling studies with a low risk of bias and high quality [23–25, 28, 30, 31], which yielded significantly larger mean differences in terms of postoperative pain scores at 6, 12 and 24 h than pooling other studies (Additional file 5). Egger’s test indicated obvious publication bias in the analysis of postoperative pain scores at 6, 12 and 24 h but no publication bias in analysis of PONV (Additional file 6). According to GARDE, there was low level of certainty of evidence for pain score at 2 and 24 h, moderate level for pain score at 6 and 12 h and postoperative opioid consumption, and high level for risk of PONV (Additional file 7).

## Discussion

SAPB is a simple and cost-effective technique of regional nerve block. SAPB combined with general anesthesia represents a promising multimodal analgesia for postoperative pain management, but its analgesic effectiveness after VATS needs confirmation. The present meta-analysis, based on 12 RCTs involving 837 participants undergoing VATS, demonstrated that perioperative SAPB could significantly reduce postoperative pains scores, postoperative opioid consumption and risk of PONV. The absolute mean differences of pain scores increased from 1.58 at 2 h to 2.07 at 6 h, and then gradually decreased to 1.74 at 12 h and 1.06 at 24 h after surgery. These changes suggested a better analgesic effectiveness of SAPB in the early phase than the late phase after VATS. Overall, the meta-analysis demonstrates that SAPB is an effective and safe adjunct to conventional general anesthesia for postoperative pain management in VATS.

There are two major injection options of SAPB, i.e. superficial SAPB and deep SAPB [15, 34]. The operators clearly identify the latissimus dorsi muscle, serratus anterior muscle and intercostal muscle using ultrasound, and then insert a needle between the latissimus dorsi and serratus anterior muscle (superficial SAPB) or between the serratus anterior and intercostal muscle (deep SAPB). Both SAPB techniques are widely used, but which one is more effective remains controversial. Theoretically, superficial SAPB may provide more extensive and longer blocking effect and is safer than deep SAPB. Moon S et al*.* conducted a RCT to compare superficial and deep SAPB after VATS lobectomy and found similar intraoperative analgesic efficacy [35]. Qiu L et al. found a stable and longer-lasting postoperative analgesic effect of superficial SAPB compared with deep SAPB [24]. Conversely, Piracha et al. suggested deep SAPB might be more efficacious than superficial SAPB for postmastectomy pain control [34]. Edwards J et al*.* observed a significantly reduced oral morphine equivalents and lower pain scores for patients undergoing mastectomy when comparing deep SAPB to superficial SAPB [36]. In the present meta-analysis, both superficial and deep SAPB were effective in postoperative pain control. Subgroup analysis showed a slightly larger reduction of pain scores at 6, 12 and 24 h in deep SAPB group than superficial SAPB group, but the difference was not statistically different (between-subgroup comparison *P* > 0.05). Therefore, more evidence needs to be accumulated for the decision making of SAPB injection strategy.

The present meta-analysis focused on the single-shot SAPB for relieving acute pain after VATS. Continuous SAPB, using a PCA device, is another effective method for postoperative acute pain control, and highly recommended for prolonged analgesia [37]. Several RCTs have demonstrated continuous SAPB has superior performance of pain relief to traditional continuous analgesia after VATS [38], thoracotomy [39] and major shoulder surgery [40]. However, the comparison between single-shot and continuous SAPB was less performed. Er J et al*.* conducted a 3-arm trial assigning patients to single-shot SAPB, continuous SAPB and PCA group [23]. They found, compared to single SAPB, continuous SAPB had higher quality of recovery scores and a lower incidence of postoperative complications, but showed higher active pain scores [23]. More investigations are still needed for the comparison between single-shot and continuous SAPB.

In addition to SAPB, there are other options for regional block, including TEA, TVPB and ICNB. TEA technique, considered as the gold standard for postoperative analgesia and used for decades in thoracic surgery, is technically more difficult for surgeons and has more complications such as accidental dural penetration, neuraxial hematoma and postoperative hypotension [41]. TVPB, injecting local analgesics into paravertebral space, also has good anesthetic and analgesic effects, which is similar to TEA [42, 43]. A recent randomized trial compared the analgesic effectiveness between TVPB, ESPB and ICNB, which favored TVPB due to a more successful analgesia and less morphine consumption [44]. Recently, several RCTs showed that SAPB conferred comparable postoperative analgesic effect to TVPB with fewer complications in VATS [45–47]. ICNB, injecting local anesthetics into multiple intercostal nerves, is effective in reducing postoperative pain, but lacks long-term analgesia and has high incidence of neuropathic pain and intercostal muscle paralysis [48]. Among patients undergoing VATS, two randomized trials showed similar postoperative analgesic effect between SAPB and ICNB [49, 50], while one trial demonstrated more effective pain relief and reduced morphine requirement of SAPB than ICNB [50]. A recent network meta-analysis incorporating 21 trials suggested SAPB and ICNB had distinct advantages [51], while another two indicated TVPB as a better option [52, 53]. Nevertheless, the best choice of regional block techniques in VATS still remains debatable.

The analgesic effectiveness of SAPB has been compared to general anesthesia in patients undergoing VATS by a previous meta-analysis [54]. However, our study has some strengths compared with the previous one. Firstly, the present one has a larger sample size as more trials were performed recently, indicating that our study has more statistical power. Secondly, we performed subgroup analysis of two major injection options of SAPB, i.e. SSAPB and DSAPB. We found no significant difference of postoperative pain scores between both subgroups, indicating a similar analgesic effectiveness of these two options. Thirdly, we performed further sensitivity analysis according risk of bias assessment and study quality. We found, in well-designed RCTs with a low risk and bias and high quality, SAPB showed a greater improvement of postoperative pain scores, which may be underestimated by adding trials with high risk of bias and low quality.

The present meta-analysis has some limitations that may weaken our conclusions. Firstly, there is substantial between-study heterogeneity, which may be due to different surgical types, anesthetic management, analgesic drugs and concentrations. Secondly, the sample sizes of each included trial and the meta-analysis are relatively small, indicating the statistical power may not be sufficient. Thirdly, the comparisons between SAPB to the other regional analgesic methods are not performed, which does not provide evidence for a better choice of regional block.

## Conclusion

In conclusion, our meta-analysis demonstrates single-shot SAPB can effectively relieve postoperative pain, lessen postoperative opioid consumption and reduce incidence of PONV. SAPB is a promising, excellent adjunct to conventional general anesthesia for postoperative pain management in patients undergoing VATS. However, these findings need further confirmation by more high-quality RCTs in the future.

## Supplementary Information

**Additional file 1.** 

**Additional file 2.** 

**Additional file 3.** 

**Additional file 4.** 

**Additional file 5.** 

**Additional file 6.** 

**Additional file 7.** 

## Data Availability

The dataset supporting the conclusions of this article are included within the article.

## Abbreviations

ESPB: Erector spinae plane block
ICNB: Intercostal nerve blockade
PCA: Patient-controlled analgesia
PONV: Postoperative nausea and vomiting
RCT: Randomized controlled trial
RR: Relative risk
SAPB: Serratus anterior plane block
TEA: Thoracic epidural analgesia
TPVB: Thoracic paravertebral blockade
VATS: Video-assisted thoracoscopic surgery
