# Supplementary material for: Analgesic effectiveness of serratus anterior plane block in patients undergoing video-assisted thoracoscopic surgery: a systematic review and updated meta-analysis of randomized controlled trials
Source: BMC Anesthesiol. 2023 Jul 13;23:235. doi: 10.1186/s12871-023-02197-8 (PMC10339549; doi:10.1186/s12871-023-02197-8)
Supplement: Supplementary file 2 — Additional file 2. [file 12871_2023_2197_MOESM2_ESM.docx]

Table S1. Search strategy for each electronic database.

| PubMed | ("serratus anterior plane block" OR "serratus plane block" OR "serratus anterior block" OR SAPB) AND (“thoracic surgery” OR thoracoscopic OR “video-assisted thoracic surgery” OR VATS) |
| --- | --- |
| EMBASE | ("serratus anterior plane block" OR "serratus plane block" OR "serratus anterior block" OR SAPB) AND (“thoracic surgery” OR thoracoscopic OR “video-assisted thoracic surgery” OR VATS) |
| Web of Science | 1# TS=(serratus anterior plane block) OR TS=(serratus plane block) OR TS=(serratus anterior block) OR TS=SAPB  2# TS=(thoracic surgery) OR TS=thoracoscopic OR TS=(video-assisted thoracic surgery) OR TS=VATS  1# AND 2# |
| Cochrane Library | ("serratus anterior plane block" OR "serratus plane block" OR "serratus anterior block" OR SAPB) AND (“thoracic surgery” OR thoracoscopic OR “video-assisted thoracic surgery” OR VATS) |
