# Supplementary figures and images for: Analgesic effectiveness of serratus anterior plane block in patients undergoing video-assisted thoracoscopic surgery: a systematic review and updated meta-analysis of randomized controlled trials
Source: BMC Anesthesiol. 2023 Jul 13;23:235. doi: 10.1186/s12871-023-02197-8 (PMC10339549; doi:10.1186/s12871-023-02197-8)

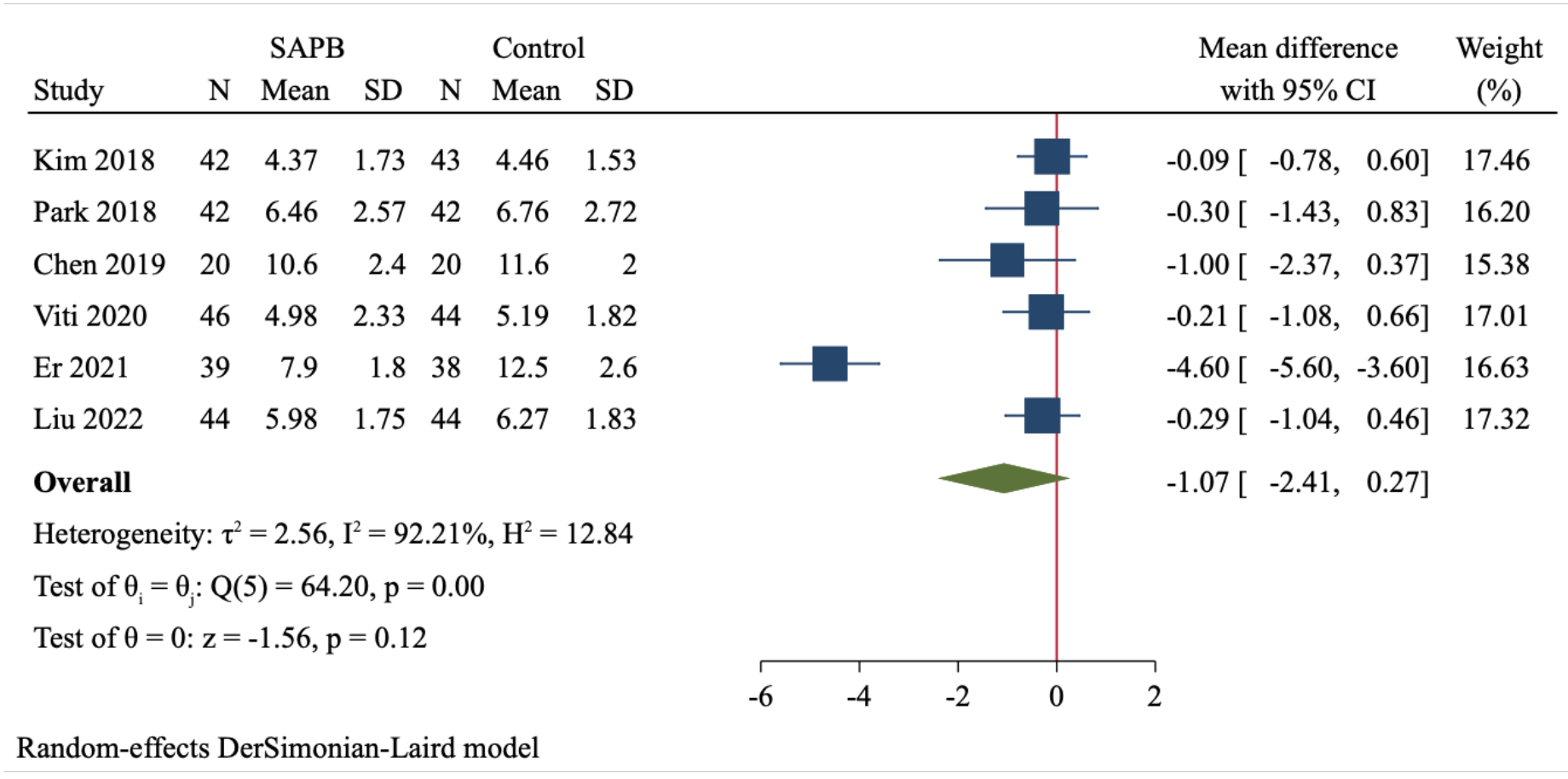


Fig. S1 Forest plot of meta-analysis for length of hospital stay

Supplement: Supplementary file 3 — Additional file 3. [file 12871_2023_2197_MOESM3_ESM.docx]

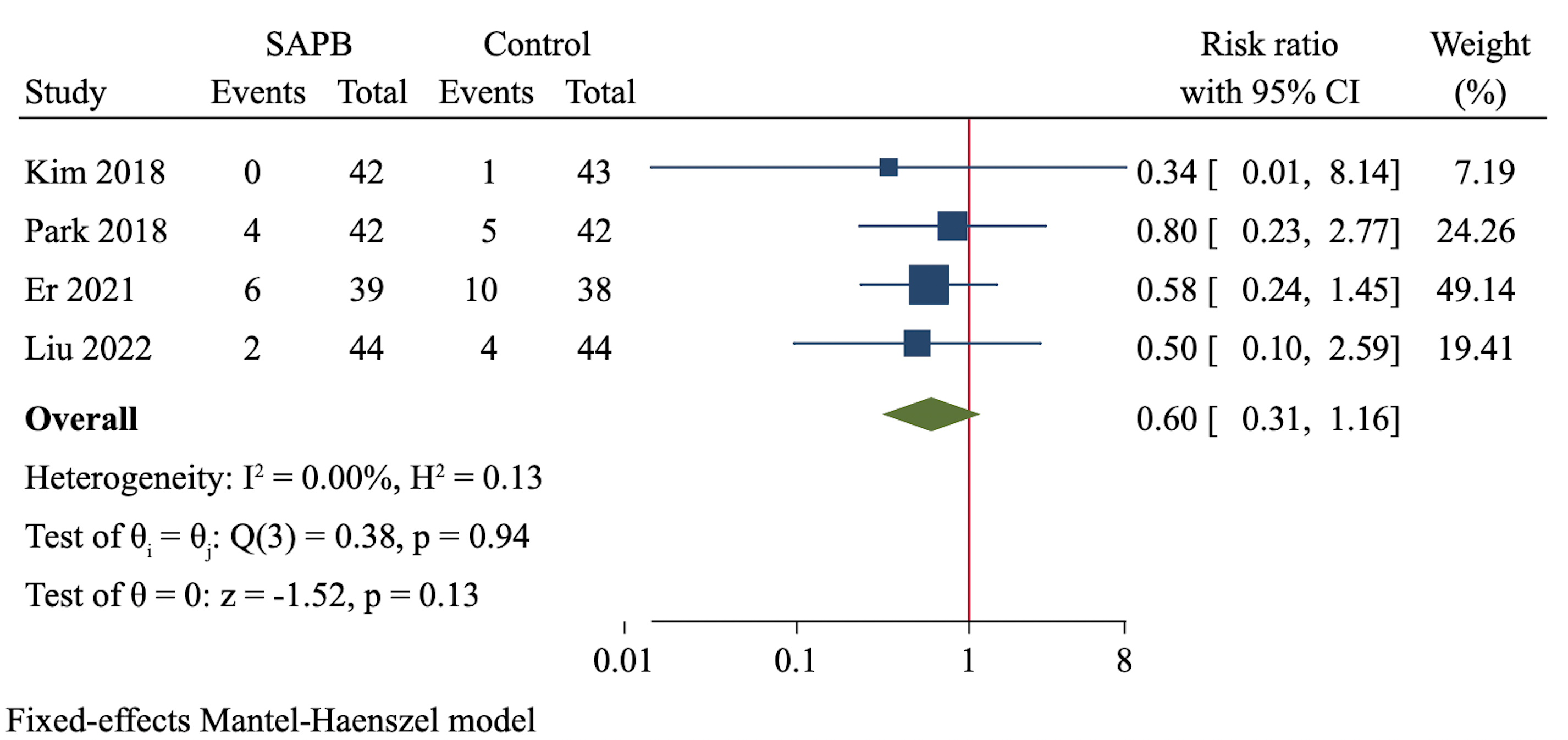


Fig. S2: Forest plot of meta-analysis for dizziness.

Supplement: Supplementary file 4 — Additional file 4. [file 12871_2023_2197_MOESM4_ESM.docx]
