# Supplementary material for: Analgesic effectiveness of serratus anterior plane block in patients undergoing video-assisted thoracoscopic surgery: a systematic review and updated meta-analysis of randomized controlled trials
Source: BMC Anesthesiol. 2023 Jul 13;23:235. doi: 10.1186/s12871-023-02197-8 (PMC10339549; doi:10.1186/s12871-023-02197-8)
Supplement: Supplementary file 5 — Additional file 5. [file 12871_2023_2197_MOESM5_ESM.docx]

Table S2. Sensitivity analysis according to risk of bias and study quality.

| Outcomes | No. of studies | *I^2^* (%) | MD | 95%CI | *P* | *P* (between subgroup comparison) |
| --- | --- | --- | --- | --- | --- | --- |
| Postoperative pain score at 6 hours | | |  |  |  | 0.010 |
| Low risk of bias and high quality | 5 | 91.3 | -2.69 | -3.62 to -1.75 | <0.001 |  |
| The others | 4 | 80.2 | -1.22 | -1.84 to -0.59 | <0.001 |  |
| Postoperative pain score at 12 hours | | |  |  |  | 0.008 |
| Low risk of bias and high quality | 4 | 95.0 | -2.73 | -4.02 to -1.44 | <0.001 |  |
| The others | 5 | 64.4 | -0.90 | -1.28 to -0.52 | <0.001 |  |
| Postoperative pain score at 24 hours | | |  |  |  | 0.072 |
| Low risk of bias and high quality | 4 | 90.6 | -1.93 | -3.32 to -0.54 | 0.007 |  |
| The others | 5 | 85.0 | -0.56 | -1.10 to -0.03 | 0.039 |  |

MD: mean difference; CI: confidence interval.
