# Supplementary material for: Analgesic effectiveness of serratus anterior plane block in patients undergoing video-assisted thoracoscopic surgery: a systematic review and updated meta-analysis of randomized controlled trials
Source: BMC Anesthesiol. 2023 Jul 13;23:235. doi: 10.1186/s12871-023-02197-8 (PMC10339549; doi:10.1186/s12871-023-02197-8)
Supplement: Supplementary file 6 — Additional file 6. [file 12871_2023_2197_MOESM6_ESM.docx]

Table S3. Egger’s test for publication bias.

| Outcome | t | *P* |
| --- | --- | --- |
| Postoperative pain scores |  |  |
| 6 hours | -1.87 | 0.061 |
| 12 hours | -1.91 | 0.057 |
| 24 hours | -2.64 | 0.008 |
| PONV | -0.79 | 0.432 |

PONV: postoperative nausea and vomiting.
