# Supplementary material for: Analgesic effectiveness of serratus anterior plane block in patients undergoing video-assisted thoracoscopic surgery: a systematic review and updated meta-analysis of randomized controlled trials
Source: BMC Anesthesiol. 2023 Jul 13;23:235. doi: 10.1186/s12871-023-02197-8 (PMC10339549; doi:10.1186/s12871-023-02197-8)
Supplement: Supplementary file 7 — Additional file 7. [file 12871_2023_2197_MOESM7_ESM.docx]

Table S4 Summary of the certainty of evidence.

| No. of studies (participants) | Risk of bias | Inconsistency | Indirectness | Imprecision | Publication bias | Certainty of evidence |
| --- | --- | --- | --- | --- | --- | --- |
| Pain score at 2h, 4 RCTs (218) | Not serious | Not serious | Not serious | Serious | None | Low |
| Pain score at 6h, 9 RCTs (631) | Not serious | Not serious | Not serious | Not serious | Suspected | Moderate |
| Pain score at 12h, 9 RCTs (606) | Not serious | Not serious | Not serious | Not serious | Suspected | Moderate |
| Pain score at 24h, 9 RCTs (606) | Not serious | Serious | Not serious | Not serious | Suspected | Low |
| Postoperative opioid consumption, 6 RCTs (450) | Not serious | Not serious | Not serious | Serious | None | Moderate |
| PONV, 11RCTs (701) | Not serious | Not serious | Not serious | Not serious | None | High |
